# Autoimmune Pancreatitis, Diabetic Ketoacidosis, and Severe Hypertriglyceridemia: Unraveling a Pathophysiological Triad

**DOI:** 10.7759/cureus.112381

**Published:** 2026-07-09

**Authors:** Deive Vinga Akoubou, Inass Chaari, Cheick Oumar Keita, Annick Abessolo, Ayoub Idrissi, Azzelarab Meftah, Hamza El Jadi

**Affiliations:** 1 Endocrinology, Diabetes and Metabolism, Avicenna Military Hospital, Marrakech, MAR; 2 Biosciences and Health Laboratory, Faculty of Medicine and Pharmacy, Cadi Ayyad University, Marrakech, MAR

**Keywords:** acute pancreatitis, autoimmune pancreatitis, diabetic ketoacidosis, hypertriglyceridemia, igg4-related disease

## Abstract

Autoimmune pancreatitis (AIP) is a rare form of chronic pancreatitis and an uncommon cause of recurrent acute pancreatitis. Although diabetes mellitus is a frequent manifestation of AIP, presentation as diabetic ketoacidosis (DKA) is exceptional. The coexistence of DKA and severe hypertriglyceridemia (HTG) in the setting of AIP represents a rare clinical association with important diagnostic and therapeutic implications. A 43-year-old man with type 2 diabetes mellitus and two previous episodes of HTG-associated acute pancreatitis presented with severe epigastric pain, persistent vomiting, and Kussmaul respiration. Laboratory investigations revealed moderate DKA (pH, 7.12; bicarbonate, 12 mmol/L), severe HTG (16.0 g/L), and markedly elevated serum lipase (600 IU/L). Contrast-enhanced abdominal CT demonstrated diffuse pancreatic enlargement with a characteristic sausage-shaped appearance and Balthazar grade E acute pancreatitis. The recurrence of pancreatitis, together with the characteristic imaging findings, prompted suspicion of type 1 AIP. Serum IgG4 levels were subsequently found to be elevated (240 mg/dL), further supporting the diagnosis in accordance with the International Consensus Diagnostic Criteria. The patient was treated with aggressive intravenous fluid resuscitation, continuous intravenous insulin infusion, and early corticosteroid therapy, resulting in rapid clinical improvement and normalization of metabolic abnormalities within 72 hours. This case illustrates a rare association between type 1 AIP, DKA, and severe HTG. We propose a biologically plausible pathophysiological interaction in which pancreatic inflammation may have contributed to transient insulin deficiency, precipitating DKA and subsequently promoting severe HTG, thereby perpetuating pancreatic injury. Although this sequence cannot be established from a single case, recognizing this uncommon presentation may facilitate earlier diagnosis and appropriate multidisciplinary management. AIP should be considered in patients presenting with recurrent pancreatitis associated with DKA, severe HTG, and characteristic pancreatic imaging findings. Early recognition and timely multidisciplinary management may improve clinical outcomes. Further studies are needed to better define the relationship between these conditions.

## Introduction

Autoimmune pancreatitis (AIP) is a distinct form of chronic pancreatitis, classified into type 1 (IgG4-related disease) and type 2 disease. The diagnosis relies on the International Consensus Diagnostic Criteria (ICDC), which integrate characteristic imaging findings, serology, histology, involvement of other organs, and response to corticosteroid therapy. Type 1 AIP predominantly affects middle-aged men and is characterized by an excellent response to corticosteroids, although disease relapse is not uncommon. Despite increasing recognition, AIP remains an uncommon cause of acute pancreatitis and is frequently misdiagnosed as pancreatic malignancy or other causes of recurrent pancreatitis [1-3].

Diabetes mellitus is a frequent endocrine manifestation of AIP, resulting from inflammatory injury to pancreatic tissue and impaired β-cell function. However, presentation as diabetic ketoacidosis (DKA) is exceptionally rare. During acute pancreatic inflammation, transient insulin deficiency may precipitate severe metabolic decompensation in susceptible individuals. This insulin-deficient state promotes accelerated lipolysis and reduced lipoprotein lipase activity, leading to marked hypertriglyceridemia (HTG). In turn, the hydrolysis of excessive triglycerides into toxic free fatty acids may further aggravate pancreatic injury, creating a biologically plausible self-perpetuating pathophysiological cycle linking pancreatic inflammation, DKA, and severe HTG [4-6].

Although each of these conditions has been individually described, their simultaneous occurrence remains exceptionally uncommon. Recognizing this unusual association is clinically important because recurrent pancreatitis accompanied by diffuse pancreatic enlargement, severe metabolic derangement, and extreme HTG should prompt clinicians to consider AIP among the differential diagnoses. Early recognition may facilitate the timely initiation of corticosteroid therapy while simultaneously addressing the metabolic abnormalities. Nevertheless, the temporal relationship between these conditions remains hypothetical and should be interpreted with caution [3,4,6,7].

We report the case of a patient presenting with this rare clinical triad, highlighting the diagnostic challenges and a biologically plausible pathophysiological interplay between autoimmune pancreatic inflammation, DKA, and severe HTG.

## Case presentation

A 43-year-old man with a history of type 2 diabetes mellitus treated with a basal-bolus insulin regimen and two previous episodes of HTG-associated acute pancreatitis presented to the emergency department with a 48-hour history of severe epigastric pain radiating to the back, persistent vomiting, and progressive asthenia. During his previous admissions, AIP had not been suspected or investigated. There was no history of alcohol misuse or gallstone disease.

On admission, the patient appeared dehydrated and exhibited Kussmaul respiration. Abdominal examination revealed marked epigastric tenderness without signs of generalized peritonitis. Initial laboratory investigations revealed marked metabolic derangement (Table 1). Blood glucose was 22.0 mmol/L. Arterial blood gas analysis demonstrated a high anion-gap metabolic acidosis with a pH of 7.12 and a serum bicarbonate level of 12 mmol/L, while urinalysis showed massive ketonuria (3+), consistent with moderate DKA. Serum lipase was markedly elevated at 600 IU/L (approximately 10 times the upper limit of normal). The serum appeared grossly lactescent, and the lipid profile demonstrated severe HTG (16.0 g/L; 1,600 mg/dL) associated with hypercholesterolemia (3.0 g/L; 299.9 mg/dL), despite chronic outpatient treatment with fenofibrate (160 mg/day), rosuvastatin (40 mg/day), and omega-3 fatty acids (3,000 mg/day). Serum potassium was 3.7 mmol/L, renal function was preserved, and inflammatory markers were moderately elevated (Table 1).

**Table 1:** Laboratory results at admission and at 72 hours following treatment initiation. Sequential laboratory results demonstrating pancreatic, hepatic, inflammatory, and metabolic parameters at admission and 72 hours after treatment initiation. Serum IgG4 was positive. Normalization of serum lipase, arterial pH, bicarbonate levels, ketonuria, triglycerides, and serum potassium was observed within 72 hours of treatment initiation. WBC: white blood cells; AST: aspartate aminotransferase; ALT: alanine aminotransferase; CRP: C-reactive protein; N/A: not available

Parameter	On admission	At 72 hours	Reference range
Blood glucose (mmol/L)	22.0	10.4	3.9–6.1
Arterial pH	7.12	7.40	7.35–7.45
Bicarbonates (mmol/L)	12	26	22–26
Ketonuria	3+	Negative	Negative
Triglycerides (g/L)	16.0	1.4	<1.5
Total cholesterol (g/L)	3	N/A	<2.0
Serum lipase (IU/L)	600	45	<60
Serum IgG4 (mg/dL)	240	Not repeated	<135
Creatinine (µmol/L)	80	69	62–115
Sodium (mmol/L)	134	140	136–145
Potassium (mmol/L)	3.7	3.8	3.5–5.0
WBC (×10³/µL)	9.8	6.4	4.0–10.0
Hemoglobin (g/dL)	12.5	13.0	13.0–17.0
Platelets (×10³/µL)	172	190	150–400
AST (IU/L)	29	32	<40
ALT (IU/L)	31	34	<40
CRP (mg/L)	60	8	<5

**Figure 1:**
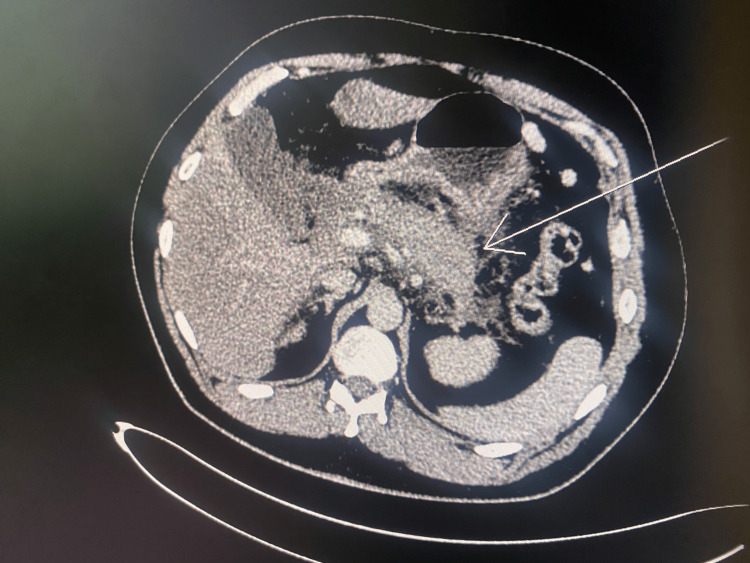
Abdominal CT scan (axial view, portal venous phase) demonstrating diffuse enlargement of the pancreas with a characteristic sausage-shaped appearance, peripancreatic fat stranding, and fluid collections consistent with Balthazar grade E acute pancreatitis. The white arrow indicates a peripancreatic fluid collection adjacent to the pancreatic tail, with surrounding inflammatory fat stranding, consistent with the extension of necroinflammation beyond the pancreatic parenchyma.

Contrast-enhanced abdominal CT demonstrated diffuse enlargement of the pancreas with the characteristic “sausage-shaped” appearance, associated with peripancreatic fat stranding and fluid collections, consistent with Balthazar grade E acute pancreatitis (Figure 1) [8].

Although severe HTG represented a plausible cause of acute pancreatitis, the recurrence of pancreatitis within a relatively short period, together with the characteristic diffuse pancreatic enlargement on CT imaging, prompted consideration of type 1 AIP. Other common causes of acute pancreatitis, including alcohol misuse and gallstone disease, were excluded based on the clinical history and imaging findings. Serum IgG4 measurement was therefore requested as part of the diagnostic workup, in accordance with the ICDC for AIP [1].

The patient was promptly managed with aggressive intravenous fluid resuscitation using isotonic saline and continuous intravenous insulin infusion. Given the strong clinical and radiological suspicion of type 1 AIP, oral prednisone was initiated at a dose of 40 mg/day within the first 24 hours of admission, before serum IgG4 results became available.

Subsequent immunological testing demonstrated an elevated serum IgG4 concentration of 240 mg/dL (reference value <135 mg/dL), which further supported the diagnosis of type 1 AIP when interpreted alongside the characteristic imaging findings and overall clinical presentation, consistent with the ICDC [1].

The patient’s clinical course was rapidly favorable. Within 72 hours, abdominal pain had completely resolved, metabolic acidosis and ketonuria had normalized, serum lipase returned to the normal range, and triglyceride levels decreased from 16.0 g/L to 1.4 g/L (Table 1). The patient was discharged in stable clinical condition and transitioned back to his pre-admission basal-bolus insulin regimen. No formal post-discharge follow-up or repeat serum IgG4 measurement was available.

## Discussion

This case illustrates the uncommon coexistence of type 1 AIP, DKA, and severe HTG, three conditions that may interact through complex metabolic and inflammatory pathways. Although diabetes mellitus is a well-recognized manifestation of AIP, presentation as DKA remains exceptionally rare, and only a limited number of similar cases have been described in the literature [4].

Based on the clinical presentation and current understanding of AIP pathophysiology, we hypothesize that pancreatic inflammation may have contributed to the transient impairment of β-cell function, thereby precipitating DKA in this patient. The resulting insulin-deficient state could subsequently have promoted uncontrolled lipolysis and reduced lipoprotein lipase activity, leading to severe HTG. In turn, excessive triglyceride hydrolysis into free fatty acids may have further aggravated pancreatic injury, creating a biologically plausible self-perpetuating pathophysiological cycle [5,6]. However, the exact temporal sequence and causal relationships cannot be established from a single case report and should therefore be interpreted with caution.

From a diagnostic perspective, severe HTG initially represented the most apparent explanation for acute pancreatitis. However, the recurrence of pancreatitis within a relatively short period, together with the characteristic diffuse “sausage-shaped” pancreatic enlargement on CT, prompted consideration of type 1 AIP. The diagnosis had not been established during the patient’s previous admissions. During the present episode, elevated serum IgG4 levels further supported the diagnosis when interpreted alongside the characteristic imaging findings and clinical context, consistent with the ICDC [1]. Although histological confirmation was not available, tissue sampling was not considered appropriate in the acute setting because of the severity of the metabolic disturbance, and the combination of clinical, radiological, and serological findings was considered sufficient to support the diagnosis.

Early recognition of AIP also had important therapeutic implications. Corticosteroid therapy was initiated within the first 24 hours of admission because of the high clinical and radiological suspicion of AIP, before serum IgG4 results became available. The subsequent elevation of serum IgG4 reinforced the diagnostic hypothesis and supported continuation of immunosuppressive therapy. This approach reflects routine clinical practice, where treatment decisions in critically ill patients frequently rely on the overall clinical picture rather than a single laboratory result.

The acute metabolic management consisted of aggressive intravenous fluid resuscitation and continuous intravenous insulin infusion, which simultaneously corrected DKA and promoted triglyceride clearance by restoring lipoprotein lipase activity. Recent evidence suggests that intensive insulin therapy may represent an effective alternative to therapeutic plasma exchange in patients with HTG-associated acute pancreatitis, particularly when DKA is the principal precipitating factor [7]. In our patient, rapid normalization of acid-base status, serum lipase, ketonuria, and triglyceride levels within 72 hours supports the effectiveness of this combined metabolic and immunosuppressive strategy (Table 1).

Long-term pancreatic dysfunction remains an important concern in patients with AIP, as both endocrine and exocrine insufficiency may persist despite resolution of the acute inflammatory episode [9]. Unfortunately, formal post-discharge follow-up, repeat serum IgG4 measurements, and assessment of pancreatic exocrine function were not available in our patient, representing a limitation of this report.

Despite these limitations, this case highlights the importance of considering AIP in patients presenting with recurrent pancreatitis associated with DKA, severe HTG, and characteristic pancreatic imaging findings. Prompt recognition of this uncommon association may facilitate timely initiation of appropriate therapy and help avoid delays in diagnosis.

## Conclusions

This case highlights the diagnostic challenge posed by the rare coexistence of type 1 AIP, DKA, and severe HTG. In patients presenting with recurrent pancreatitis associated with marked metabolic derangement and characteristic pancreatic imaging findings, AIP should be considered among the differential diagnoses. Although the proposed pathophysiological interaction remains hypothetical, it is biologically plausible and may contribute to understanding this unusual clinical presentation. Early recognition and a multidisciplinary approach, combining metabolic stabilization with timely immunosuppressive therapy when clinically appropriate, may improve patient outcomes. Additional studies are needed to further clarify the relationship between these conditions and to better define their optimal management.
